# CGHpower: exploring sample size calculations for chromosomal copy number experiments

**DOI:** 10.1186/1471-2105-11-331

**Published:** 2010-06-17

**Authors:** Ilari Scheinin, José A Ferreira, Sakari Knuutila, Gerrit A Meijer, Mark A van de Wiel, Bauke Ylstra

**Affiliations:** 1 Department of Pathology, VU University Medical Center, Amsterdam, The Netherlands; 2 Department of Pathology, Haartman Institute and HUSLAB, University of Helsinki and Helsinki University Central Hospital, Finland; 3 FIMM Technology Centre, Institute for Molecular Medicine Finland (FIMM), University of Helsinki, Finland; 4 Department of Epidemiology and Biostatistics, VU University Medical Center, Amsterdam, The Netherlands; 5 Department of Mathematics, Vrije Universiteit, Amsterdam, The Netherlands

## Abstract

**Background:**

Determining a suitable sample size is an important step in the planning of microarray experiments. Increasing the number of arrays gives more statistical power, but adds to the total cost of the experiment. Several approaches for sample size determination have been developed for expression array studies, but so far none has been proposed for array comparative genomic hybridization (aCGH).

**Results:**

Here we explore power calculations for aCGH experiments comparing two groups. In a pilot experiment CGHpower estimates the biological diversity between groups and provides a statistical framework for estimating average power as a function of sample size. As the method requires pilot data, it can be used either in the planning stage of larger studies or in estimating the power achieved in past experiments.

**Conclusions:**

The proposed method relies on certain assumptions. According to our evaluation with public and simulated data sets, they do not always hold true. Violation of the assumptions typically leads to unreliable sample size estimates. Despite its limitations, this method is, at least to our knowledge, the only one currently available for performing sample size calculations in the context of aCGH. Moreover, the implementation of the method provides diagnostic plots that allow critical assessment of the assumptions on which it is based and hence on the feasibility and reliability of the sample size calculations in each case.

The CGHpower web application and the program outputs from evaluation data sets can be freely accessed at http://www.cangem.org/cghpower/

## Background

Array comparative genomic hybridization (aCGH) is a technique that uses microarrays to perform high-resolution and genome-wide screening of DNA copy number changes. Its most important applications are in cancer research [1] and clinical genetics [2]. In this paper we focus on aCGH experiments comparing two groups of cancer samples. Previously, we introduced the Wilcoxon test with ties to identify chromosomal copy number differences when comparing two groups [3]. The goal of comparing two groups is generally to identify disease biomarkers, chromosomal regions (or genes therein) for survival, therapy, progression, *et cetera*. An important problem that arises in the planning of aCGH experiments is the choice of the sample size, which we explore here. Data analysis of microarray experiments comparing two groups generally involves calculating a test statistic for each array element and setting a cutoff for rejecting the null hypothesis of no difference between the groups. With a single array element, there are therefore two typical errors that can occur in the process. A type I error occurs when the null hypothesis is rejected even though it was actually true and the cut-off was exceeded only by chance. A type II error involves accepting a null hypothesis that should have been rejected, thus failing to identify a true difference. To broaden the perspective from individual array elements to the framework of multiple testing covering the entire microarray, two concepts are used: false discovery rate (FDR) [4] and average power. FDR is the expected percentage of discoveries that are false. Statistical power is the probability of recognizing a single array element with a true difference, and average power refers to the expected percentage of true positives that is identified. In general, it is desirable to have the FDR as close to zero and average power as close to one as possible. Setting the cut-off for rejecting the null hypothesis is a delicate balance between sensitivity and specificity; while a stringent cut-off lowers the FDR, it also lowers average power and *vice *versa.The only way to improve both, or one without affecting the other, is to increase the number of biological replicates and thus perform more arrays. Sample size calculations can generally be divided into two categories. The first category asks the user to define values for certain parameters, such as the effect size (fold change of a differentially expressed gene) and the proportion of genes that are truly differentially expressed [5-9]. The second category estimates these parameters from existing data [10,11]. The method proposed here follows the latter approach and therefore requires pilot data.

To adapt mRNA expression array power calculations for aCGH and copy number changes, two key aspects need to be taken into account. Instead of concentrations of individual mRNA molecules, the underlying biology measured by aCGH consists of blocks of chromosomal DNA. Each block is (presumably) present in a normal copy number of two, but may contain areas of one or two-copy losses and one or more gains. Higher level amplifications can also be present. The aberrations contain both driver and passenger genes, and the breakpoints may vary from one sample to another.

As the entity being measured is DNA present in a discrete number of copies (0, 1, 2, 3, 4, ...), but individual array elements yield log_2 _ratios, aCGH data preprocessing generally involves the following steps that aim to better capture the biological relevance. *Normalization *first removes technical artifacts and makes the log_2 _ratios comparable across different hybridizations. *Segmentation *then identifies areas that share a common copy number and are separated by breakpoints. Finally, *calling *determines a discrete copy number level for each segment. At the moment, there is no clear consensus regarding the optimal stage of preprocessing from which the data should be used for downstream analysis. We discussed the topic and proposed that in most cases the recommended choice be to use calls, which have the clear advantage of having an attached biological meaning [12]. For power calculations however, the use of calls is problematic, as it would require the use of the chi-square test, for which no method of sample size calculation in large FDR-based multiple testing contexts is presently available. While both normalized and segmented log ratios allow the use of a t-test, they fail to take full advantage of the adjacency of consecutive array elements. Aberrations typically show great variation in their sizes ranging from focal amplifications to gains and losses of entire chromosome arms. Working directly with the original array elements does not take this into account, and gives larger aberrations significantly more weight than smaller ones as they contain more array elements. A possible improvement is therefore to replace array elements with regions, which are defined as a series of neighboring array elements sharing the same copy number signature. This reduces dimensionality with little loss of information [13]. Throughout this paper, the term *regions *is used to refer to the results of this analysis step.

For CGHpower, we are combining the advantages of regions with the feasibility of log ratios, by replacing the hard calls with median log ratios of all the array elements within a region. Together with these region-wise log ratios (RWLRs), the regions are then taken as a representation of the underlying biology (*i.e. *chromosomal regions with varying copy number levels). Each region is coupled to a null hypothesis stating that the means of the two groups do not differ from each other, which is the framework required for the power calculations proposed here. Regions that have a true difference between the two groups (generally normal copy number in one group and a gain, loss or amplification in the other) will be referred to as "differentially behaving regions".

After this preprocessing, power calculations are performed using regions as Ferreira *et al. *[14] previously described for both real and simulated gene expression data. T-statistics and p-values are calculated for each region from the RWLRs. All p-values from non-differentially behaving regions are expected to follow a uniform distribution, while those from the differentially behaving ones should follow another, unknown distribution (*G*). Two separate estimators of *G *are calculated: a non-parametric () and a parametric one (Ĝ_n_), which assumes that *G *follows a normal distribution. Both of these estimators depend on another unknown parameter, *γ*, which is the proportion of non-differentially behaving regions. When the estimate of *γ *used to calculate Ĝ_*n *_and  moves away from its true value, the difference between the two *G *estimators increases. The estimate of *γ *is therefore chosen so that this difference is minimized. The limiting density of effect sizes (λ) is then estimated using deconvolution, and so is *G*. Once these estimates have been calculated, approximate sample size calculations can be made using an adaptive version of the Benjamini-Hochberg method for multiple testing. While the original method [4] allows control over the FDR, the adaptive version also allows the estimation of average power [10].

While optimizing the protocol, there were certain options that we considered: whether to calculate the RWLRs as the mean or median of the log ratios, whether to use the Student's t-test assuming equal variances or Welch's t-test that allows unequal variances, and finally whether to calculate the p-values from normal or Student's t-distribution. All of the possible combinations were tested, and the optimum performance was observed with median log ratios, unequal variances and the normal distribution. These choices were then fixed in CGHpower.

## Implementation

### Evaluation Data Sets

To evaluate the performance of CGHpower, eight recently published aCGH data sets that could be divided into two groups were collected. They will be referred to as Chin *et al. *[15], Douglas *et al. *[16], Fridlyand *et al. *[17], Myllykangas *et al. *[18], Nymark *et al. *[19], Postma *et al. *[20], Smeets *et al. *[21] and Wrage *et al. *[22] A total of five different array types were used among the data sets: VUmc 30 K spotted oligo [23] for data sets [15,20,22], Agilent Human 1 cDNA Microarray for [18,19], 3 K BAC array [24] for [16], 2 K BAC array [25] for [17] and 6 K BAC array for [21]. Table 1 provides a summary of the cancer and array types, together with group definitions and sizes.

**Table 1:** Evaluation data sets

Data Set	Array Type	Probes	Regions	Cancer Type	Groups (Samples)
Chin *et al.*	spotted oligo	26,755	223	breast	ER+ (113) *vs. *ER- (57)
Douglas *et al.*	BAC	3,032	142	colorectal	MSI (7) *vs. *CIN (30)
Fridlyand *et al.*	BAC	1,877	231	breast	TP53+ (10) *vs. *TP53- (52)
Myllykangas *et al.*	cDNA	11,342	260	gastric	diffuse (15) *vs. *intestinal (23)
Nymark *et al.*	cDNA	10,953	242	lung	asbestos-exposed (11) *vs. *non-exposed (9)
Postma *et al.*	spotted oligo	26,755	111	colorectal	good (16) *vs. *bad response (16)
Smeets *et al.*	BAC	4,196	143	head and neck	HPV+ (12) *vs. *HPV- (12)
Wrage *et al.*	spotted oligo	25,549	23	lung	BM+ (13) *vs. *BM- (15)
Simulation 0	in-situ oligo	42,331	440		(15) *vs. *(15)
Simulation 5	in-situ oligo	42,331	489		(15) *vs. *(15)
Simulation 10	in-situ oligo	42,331	525		(15) *vs. *(15)

Eight public data sets were collected to evaluate the performance of CGHpower. They represented five different cancer types and BAC, cDNA and oligo-based microarray platforms, with resolutions varying from 2 K to 27 K array elements. The last column contains the distinguishing factor used to divide the data set into two groups, along with the number of arrays in each group. The simulated data sets were generated by introducing artificial aberrations into a set of clinical genetics samples. A total of 11 simulations were generated, and the remaining ones are available at http://www.cangem.org/cghpower/. ER = estrogen receptor, MSI = microsatellite instability, CIN = chromosomal instability, HPV = human papilloma virus, BM = bone marrow metastasis.

### Simulated Data Sets

In addition to real data sets, evaluation was also performed with simulated data. While generating the simulations, we attempted to implement realistic aspects of both signal and noise of tumor profiles. In the context of an aCGH experiment comparing two groups, the signal consists of aberrant regions that are specific to one of the groups. Noise consists of regions common to both groups, random aberrations in individual samples, and technical noise. Further characteristics are also that the sizes of the aberrant regions vary from entire chromosomes to focal aberrations, the exact start and end positions of a region vary slightly from one sample to another, and even a "common" region might not be be present in all of the samples.

The simulated data were generated by introducing artificial aberrations into a data set of clinical genetics samples of patients with mental retardation and no or few chromosomal aberrations [26]. To achieve a simulated data set of the desired size, resampling was performed with replacement. Aberrant regions were then randomly introduced as follows. A single array element was chosen at random as the starting point of a region. The size of the region was then chosen at random with a 10% probability for a single cytoband, 30% for three consecutive bands, 30% for six consecutive bands, 20% for the whole chromosome arm, and 10% for the entire chromosome. The type of the aberration was randomly chosen as a gain or loss with equal probabilities, but for the smallest aberrations of individual cytobands, a 2% probability for amplifications was also included. When introducing a region to a set of samples, the exact samples receiving the aberration were sampled from the Bernoulli distribution with p = 70%. Randomness was also introduced to the exact start and end positions of aberrations in individual samples by shifting the starting and ending array elements by a random number between -10 and 10.

A simulated data set of 15 + 15 arrays was generated with 30 common regions, and 5 regions for each individual sample. These copy number changes do not separate the two groups from each other, and therefore represent background noise. This data set is referred to as Simulation 0. Single regions specific to the two groups were then introduced to Simulation 0 yielding data set Simulation 1. This process was repeated ten times resulting in a set of 11 simulations with the amount of differential signal ranging from none in Simulation 0 to 10 regions specific to each group in Simulation 10. Only Simulations 0, 5 and 10 are presented in this paper, but the full CGHpower outputs for all of them are available on the program's web page.

### Preprocessing

All evaluation data sets were preprocessed starting from raw log_2 _ratios. First, the data were median normalized. Wavy patterns typically seen in many aCGH profiles were removed [26] from the 30 K arrays [15,20,22]. Normalized log ratios were segmented using the DNAcopy algorithm [27] and called by CGHcall [28] to identify gains, losses and amplifications. Regions between breakpoints were then collapsed into single data points, when shared between most of the samples [13]. Finally, the median log ratio was calculated for each of these regions in each sample, resulting in region-wise log ratios (RWLRs). All algorithms were run with default parameters, and sex chromosomes were excluded from the data.

### Sample Size Calculations

For each region, t-statistics were calculated with a Welch's t-test allowing unequal variances and p-values computed from the normal distribution. The proportion of non-differentially behaving regions (γ) was estimated by minimizing the difference between parametric (Ĝ_n_) and non-parametric () estimators of *G*, which is the unknown distribution of the p-values from differentially behaving regions. The limiting density of effect sizes (λ)and *G *were then estimated using deconvolution. Finally, with FDR fixed at 10%, these parameter estimates were used to approximate average power as a function of sample size.

## Results and Discussion

**Figure 1:**
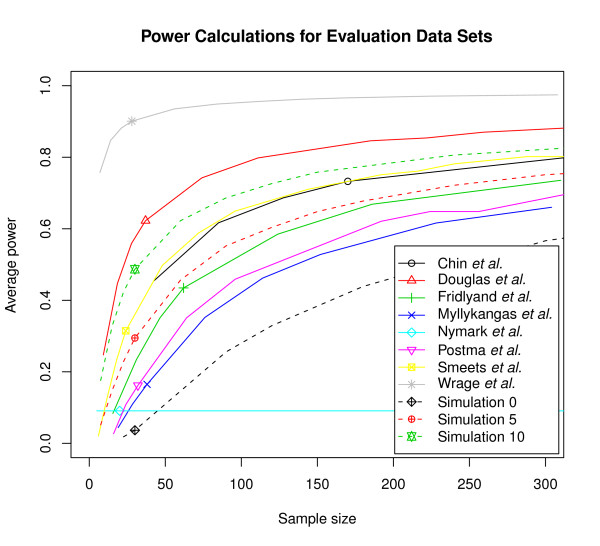
**Power calculations for evaluation data sets**. Average power estimated as a function of sample size for the eight evaluation data sets and three simulations. False discovery rate was fixed at 10%. The horizontal position of the small symbols mark the actual size of the data set that was used to calculate the estimates in each case. Real data sets are shown with solid lines and three of the simulations with dotted lines. Additional simulations are available at http://www.cangem.org/cghpower/.

Estimates of average power as a function of sample size were calculated for the eight evaluation data sets and 11 simulations (Figure 1). The reliability of the power calculations depends directly on the the quality of parameter estimation, which in turns depends on compliance with required assumptions. The first assumption is that the proportion γ of non-differentially behaving regions be "substantially" smaller than 1 *(e.g. *~0.9 will typically do, but 0.99 will not). The second assumption is that the RWLRs be approximately normally distributed, being neither particularly asymmetric (skewness) nor heavily tailed or extremely peaky (kurtosis). The complete CGHpower program output contains diagnostic plots from different stages of the power calculations procedure. These plots help determine to which extent these assumptions are fulfilled. While it is impossible to know what the true values of γ and λ are, one can easily evaluate how well the two estimators of *G *agree with each other (the "goodness-of-fit"). If they show a clear discrepancy, the accuracy of parameter estimation is questionable and the resulting power calculations consequently unreliable. Different scenarios in the quality of parameter estimation observed with the evaluation data sets are examined for each of the data sets to estimate the reliability of the calculated power.

**Figure 2:**
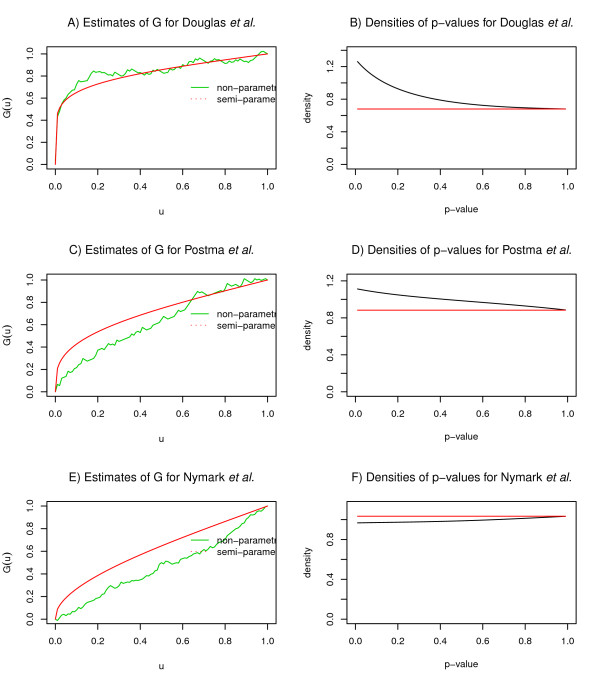
**Diagnostic plots**. The goodness-of-fit of the two estimators of *G *and densities of p-values for three data sets illustrating different scenarios in the performance of CGHpower. The data set of Douglas *et al. *shows A) a satisfactory goodness-of-fit following from B) a convex p-value density function. Mediocre operation is demonstrated with the data set of Postma *et al. *C) An inferior fit results from D) a p-value density which shows a slight increase for small values, but is not convex as expected. Nymark *et al. *represents failed execution. E) The disagreement between the *G *estimators is slightly more severe and the estimated power curve is a flat line (Figure 1). F) P-values exhibit even less density at low values than would be expected by chance. In such circumstances, it is recommended that data preprocessing be carried out before uploading and only the power calculations part be performed in CGHpower.

The data sets Douglas *et al.*, Smeets *et al.*, Fridlyand *et al. *and Chin *et al. *are examples where the goodness-of-fit of the *G *estimators was satisfactory, ranked in this order according to their fits (Figure 2A). What appears to be the most important factor distinguishing these data sets from the others, is the density of the p-values. If there is no difference detected between two groups, p-values are expected to follow a uniform distribution, and their density function appears as a flat line. When the number of differentially behaving regions increases (γ moves away from 1), density at low p-values increases and the function is expected to be convex (Figure 2B). This can also be seen on the simulations where the amount of differential signal gradually increases from Simulation 0 to Simulation 10. Along with the increase in density for low p-values, also the goodness-of-fit systematically improves (data and figures at http://www.cangem.org/cghpower/).

Less satisfactory performance was observed with data sets of Postma *et al. *and Myllykangas *et al. *The goodness-of-fit shows more disagreement between the two estimators of *G *(Figure 2C) and as a result power estimates are less reliable. The density is increasing for low p-values, but slightly less and the function is not convex as expected (Figure 2D). Compared to Simulation 0, which has no true differences between the groups, the increase in p-value density for the data set of Myllykangas *et al. *is very small. One explanation is that there is simply not enough differential signal that is detectable with a t-test. Alternatively, the number of differentially behaving regions might be too low ( *i.e. γ *is too close to 1). While these data sets do give *γ *estimates of 0.75 and 0.55, respectively, these estimates cannot be trusted if the estimates of *G *disagree with each other. Therefore it is recommended that the goodness-of-fit plot be used to assess the reliability of the estimates of other parameters. Also, judging from the results with the simulated data sets, CGHpower seems to underestimate the true value of *γ*.

While assumptions regarding *γ *seem to be most important, the RWLRs are also assumed to be normally distributed. The program output contains histograms of the skewness (asymmetry) and kurtosis (peakedness) of the RWLRs, superimposed with those of a normal distribution (data on the CGHpower web page). Assumptions of normality become more critical with small sample sizes and less important with large ones. Within the evaluation data sets, most violations of normality were observed with the Chin *et al. *data set, yet this is one of the better-performing ones in terms of goodness-of-fit. This might be explained by the relatively large sample size (170) of the study. Another factor besides the number of arrays, is the number of regions found after the preprocessing step. The larger the number of regions, the better the performance of the parameter estimation and therefore the reliability of power calculations. The assumption of normality is therefore more crucial with samples containing very few biological differences.

The data sets of Nymark *et al. *and Wrage *et al. *are examples where our method failed to work, despite the differences reported and technically as well as biologically validated. In the case of Nymark *et al. *the obtained power curve is a flat line (Figure 1). This can happen when parameter estimation fails. The explanation can be found from the density of the p-values, but now the assumptions were violated more severely than in the cases of Postma *et al. *and Myllykangas *et al. *The density function is actually concave and shows even less density at low p-values than would be expected by chance (Figure 2F). With Wrage *et al.*, failure can be observed at the preprocessing step, as only 23 regions are detected (Table 1). Since the sex chromosomes are excluded from the analysis, this means that only one copy number breakpoint was detected in the whole genome using the fixed CGHpower preprocessing described above. As preprocessing and power calculations procedures are fixed earlier in CGHpower, it was not optimized it for every aCGH platform or data set. Allowing the user to fine-tune different settings and immediately see the result of each change would require implementing a more complex user interface, similar to desktop software, which would be impractical for a single-purpose web tool. As an alternative option, if the goodness-of-fit and density plots indicate that power calculations failed, users can perform preprocessing independently, turn off the preprocessing step from the program, and perform the power calculations only.

### Consistency as the Pilot Size Is Increased

**Figure 3:**
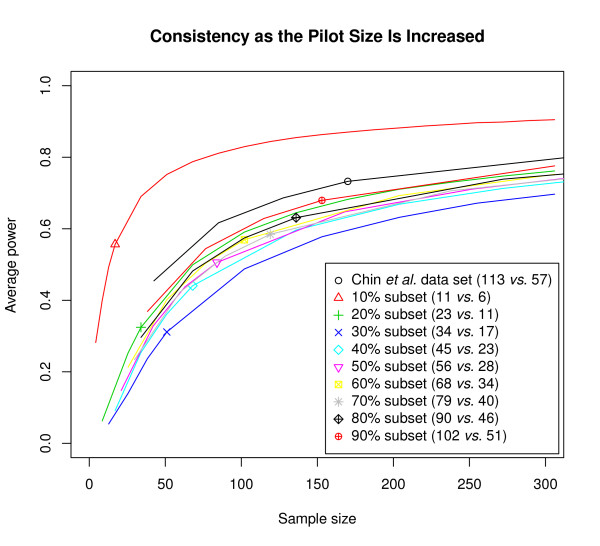
**Consistency as the pilot size is increased**. To evaluate whether power estimates obtained from smaller pilots are in fact representative of larger data sets, the calculations were performed with subsets of the Chin *et al. *data. Resampling without replacement was used to obtain subsets from 10% to 90% of the original data set. Each resampling was repeated ten times and results averaged. The horizontal position of the small symbols mark the size of the subset used to obtain each power estimate.

CGHpower was initially developed to be used on smaller pilot data sets in the planning stages of larger microarray experiments or for verifying power achieved in past experiments. We wanted to evaluate whether the resulting power estimates hold while more and more arrays are added to the data set. Assuming that a pilot of 10 + 10 arrays has estimated an experiment with 40 + 40 arrays should result in an average power of approximately 70%. The data set of 80 arrays is then generated and for verification the power calculations are repeated with the entire data set. If the new results indicate that the achieved power is in fact only 50%, and that 20 + 20 new arrays are needed in order to achieve our goal of 70%, then the two power calculations have to be declared inconsistent. To evaluate whether the power estimates remain consistent while the pilot size is increased, power was calculated with smaller subsets of the Chin *et al. *data set, since it is our largest one. This data set contains a total of 170 arrays (113 *vs. *57), which was split into smaller subsets to represent pilots of a larger study. Nine resamplings ranging from 10% (11 *vs. *6 arrays) to 90% (102 *vs. *51) of the original data set were randomly selected for the power calculations. Each resampling was repeated 10 times and the results were averaged. Two of the ten repetitions of the 10% subset and one repetition in the 20% subset experienced a failed power estimation resulting in flat power curves as with the Nymark *et al. *data set. These cases were removed before averaging the results. A plot of the resulting power estimates shows that except for the smallest subset (11 *vs. *6 arrays), the results appear to be consistent (Figure 3). This suggests that as long as the pilot is of sufficient size, power estimates generated with CGHpower using smaller pilot data sets are in fact representative of a subsequent larger study. While the exact requirement for a "sufficient pilot" is hard to define beforehand, the power calculations can be repeated when more arrays are performed to see whether power estimates are still changing or have been stabilized.

## Conclusions

We have explored sample size calculations in the context of aCGH and copy number changes and propose a dedicated tool for this purpose. From a pilot data set, CGHpower estimates the biological diversity between two groups of cancer samples and estimates average power as a function of sample size using an adaptive version of the Benjamini-Hochberg method for multiple testing [4,10]. Pilot data is used for parameter estimation and this requires certain assumptions to hold in an approximate sense. We have evaluated the performance of CGHpower with eight published data sets, four of which show satisfactory performance using predefined preprocessing measures. Among these data sets were BAC and oligo-based array platforms, whose resolution varied from less than 2 K for BACs to almost 27 K for oligos. The differences in resolution did not have a direct impact on the obtained power estimates, which should be determined more by the amount of biological variation between the two groups.

In two data sets violations of critical assumptions lead to problems in parameter estimation and therefore power estimates are less reliable. More severe violations and/or the inflexibility of a completely predefined analysis procedure lead to failed execution for the two other data sets. Even though the proposed method has its limitations, it is to our knowledge the only proposed one for aCGH data and copy number changes. As the program allows performance evaluation through diagnostic plots, critical judgement can be applied for each data set.

As a summary on the evaluation of CGHpower results, users should consider paying attention to the following: 1) Do the copy number profile plots appear similar to the aberrations that you have detected in your own analysis? If CGHpower does not seem to detect the important aberrations, consider performing the preprocessing before uploading and use CGHpower only for the power calculations. 2) Do the estimators of *G *agree with each other? If the goodness-of-fit is poor, so will other parameter (and resulting power) estimates. 3) Is the density function of the p-values convex, and showing a higher density at small p-values? A straight or concave function might be caused by too small effect size, or γ being too close to one. 4) Excess skewness and/or kurtosis in the data might also affect the performance, but this seems to be less crucial.

The proposed method uses log ratios instead of calls, even though we feel the latter is generally the preferred choice when working with aCGH data. Calls have the benefit of a clear biological meaning and are therefore easier to interpret. However, their use for power calculations in the context of FDR is problematic, as it would require using the chi-square test, a setting that is not as well developed as the Gaussian one. Also, as log ratios are the basis for calls in the first place, they do contain all the necessary information even though they are not as clear to interpret.

In comparison to sample size calculations for mRNA expression arrays, the differentiating factor for aCGH studies is the concept of regions, which stems from the different biological phenomenon underlying the microarray log_2 _ratios. Compared to the number of array elements, the number of regions is relatively small, which presents challenges to parameter estimation from the data. As the total number of regions is remarkably smaller than with expression arrays, the estimation might fail if the number of differentially behaving regions is too small, even if there is a true difference between the groups.

An important concern when performing power calculations is the actual power requirement. A power curve typically plateaus out at some point, indicating saturation. Increasing the average power from *e.g. *60% to 70% requires a significantly bigger increase in sample size than is needed for an increase from 50% to 60%. Therefore it is difficult to set a a predefined gold standard of adequate power. One option is to try to find where the slope of the power curve is decreasing rapidly. This should give a reasonable compromise between statistical power and cost of the experiment. Another aspect worth pointing out, is that the level of power needed also depends on the research question. For example, if the goal is to construct a classifier that can classify future samples into one of the two groups, a lower level of average power might yield a perfectly satisfactory classifier even though not all differences are detected.

## Availability and requirements

CGHpower is a web-based application and can be freely accessed at http://www.cangem.org/cghpower/. It allows direct uploads and can also automatically retrieve data stored in the CanGEM database [29]. The computation times of CGHpower may vary considerably depending on the number of samples and array elements in the data set, and also on the prevailing load of the Linux cluster where the calculations are performed. As an example, running times for a data set of 30 samples and 42 K array elements have been around 1-1.5 hours in our test runs. The software has been implemented in R [30] and the source code is available upon request.

## Authors' contributions

BY conceived the study. IS, JAF, MAW and BY designed CGHpower. IS performed the implementation. IS, JAF, MAW and BY wrote the manuscript with critical comments from SK and GAM. All authors read and approved the final manuscript.
